# Commissioning and validation of RayStation treatment planning system for CyberKnife M6

**DOI:** 10.1002/acm2.13732

**Published:** 2022-07-20

**Authors:** Maude Gondré, Mireille Conrad, Véronique Vallet, Jean Bourhis, François Bochud, Raphaël Moeckli

**Affiliations:** ^1^ Institute of Radiation Physics Lausanne University Hospital and Lausanne University Lausanne Switzerland; ^2^ Radio‐Oncology Department Lausanne University Hospital and Lausanne University Lausanne Switzerland

**Keywords:** collapsed cone, commissioning, CyberKnife, Monte Carlo, treatment planning system, validation measurements

## Abstract

**Background:**

RaySearch (AB, Stockholm) has released a module for CyberKnife (CK) planning within its RayStation (RS) treatment planning system (TPS).

**Purpose:**

To create and validate beam models of fixed, Iris, and multileaf collimators (MLC) of the CK M6 for Monte Carlo (MC) and collapsed cone (CC) algorithms in the RS TPS.

**Methods:**

Measurements needed for the creation of the beam models were performed in a water tank with a stereotactic PTW 60018 diode. Both CC and MC models were optimized in RS by minimizing the differences between the measured and computed profiles and percentage depth doses. The models were then validated by comparing dose from the plans created in RS with both single and multiple beams in different phantom conditions with the corresponding measured dose. Irregular field shapes and off‐axis beams were also tested for the MLC. Validation measurements were performed using an A1SL ionization chamber, EBT3 Gafchromic films, and a PTW 1000 SRS detector. Finally, patient‐specific QAs with gamma criteria of 3%/1 mm were performed for each model.

**Results:**

The models were created in a straightforward manner with efficient tools available in RS. The differences between computed and measured doses were within ±1% for most of the configurations tested and reached a maximum of 3.2% for measurements at a depth of 19.5‐cm. With respect to all collimators and algorithms, the maximum averaged dose difference was 0.8% when considering absolute dose measurements on the central axis. The patient‐specific QAs led to a mean result of 98% of points fulfilling gamma criteria.

**Conclusions:**

We created both CC and MC models for fixed, Iris, and MLC collimators in RS. The dose differences for all collimators and algorithms were within ±1%, except for depths larger than 9 cm. This allowed us to validate both models for clinical use.

## 1 INTRODUCTION

Designed to deliver stereotactic treatments, the CyberKnife (CK) M6 device (Accuray, USA) allows the use of fixed, Iris, and multileaf collimators (MLC). The fixed and Iris collimators provide field diameters of 5–60 mm with circular and dodecahedral apertures, respectively. The MLC offers the possibility of shaping irregular fields with a maximum aperture of 115 × 100 mm^2^. Until now, the only available treatment planning system (TPS) for CK optimization was the Precision (Accuray, USA), with RayTracing and Monte Carlo (MC) calculation algorithms. Recently, a module for CK planning (called RayCK) with collapsed cone (CC) and MC algorithms was introduced in version 11A of the TPS RayStation (RS) (RaySearch Laboratories, Sweden). The CC algorithm is based on the convolution superposition method and is known to have great accuracy in heterogeneous conditions as it accounts for lateral energy transport without assuming a homogeneous medium.^1^ The MC algorithm is a Class II algorithm (condensed history)^1^ that calculates dose‐to‐medium as recommended by the AAPM TG 329.^2^ The objective of this work is to present the modeling and validation of the RS TPS for fixed, Iris, and MLC collimators of the CK M6.

## 2 MATERIALS AND METHODS

### 2.1 Collapsed cone and Monte Carlo modeling in RayStation

#### 2.1.1 Measurements for modeling

We performed commissioning measurements with a PTW 60018 stereotactic diode (PTW, Germany) in a water tank with a fixed source–skin distance of 785 mm and a reference depth of 15 mm. A semi‐flex 31010 ionization chamber (PTW, Germany) was used as reference chamber to reduce fluctuations during the measurements.

The measurements needed to build the model were specified in the Beam Commissioning Data Specification of RS^2^ and are made of output factors (OF), left–right (LR) and target–gun (TG) profiles at depths of 15, 50, 100, and 300 mm, as well as percentage depth doses (PDDs) on the central axis (CAX). All fixed and Iris diameters were measured, except the Iris 5‐mm diameter that is not supported by RS because of its potential mechanical instability. For the MLC, we measured 10 square fields of 7.6 × 7.7 mm^2^ to 100.0 × 100.1 mm^2^ and a rectangular field of 115.0 × 100.1 mm^2^. For the Iris collimator, due to its dodecahedral shape aperture, profiles with angles of 15 and 105 degrees were acquired in addition to LR (0 degree) and TG (90 degrees) profiles. The four acquired profiles were averaged to obtain the profile imported into RS. For the MLC, a diagonal profile was required for the largest field size of 115.0 × 100.0 mm^2^ in order to correctly model the field corners. The OF for all the collimators were measured at 15‐mm depth and were normalized with the value obtained with the 60‐mm fixed collimator. As recommended by IAEA TRS 483,^3^ the OFs of beams of diameter below 20 mm were corrected by a factor $k_{Q_{\mathrm{clin}},\;Q_{\mathrm{msr}}\;}^{f_{\mathrm{clin}},\;f_{\mathrm{msr}}}$ ^4^, ^5^, ^6^ where *f* and *Q* are, respectively, the clinical field size and beam quality of a non‐reference field (*f*_clin_, *Q*_clin_) and of the machine specific reference field (*f*_msr_, *Q*_msr_), which is the fixed 60‐mm diameter. The corrected OF $\Omega_{Q_{\mathrm{clin}},\;Q_{\mathrm{msr}}}^{f_{\mathrm{clin}},\;f_{\mathrm{msr}}}$ were obtained by the relation: $\Omega_{Q_{\mathrm{clin}},\;Q_{\mathrm{msr}}}^{f_{\mathrm{clin}},\;f_{\mathrm{msr}}}=k_{Q_{\mathrm{clin}},\;Q_{\mathrm{msr}}\;}^{f_{\mathrm{clin}},\;f_{\mathrm{msr}}}\cdot {\mathrm{OF}}_{\det}^{f_{\mathrm{clin}},\;f_{\mathrm{msr}}}$.

#### 2.1.2 Modeling in RayStation

The modeling was performed in the RayPhysics module of RS where several parameters were optimized to increase the matching between measured and computed curves (PDDs and profiles). Table 1 summarizes these parameters and specifies their impact on PDDs or profiles and at which collimator they were related. A flattening filter source parameter was available although there is no flattening filter in the CK. This parameter artificially helped to shape the out‐of‐field dose. A special care was taken to keep its weight low. In addition, the optimization method used (manual or auto‐modeling) is indicated. When a parameter was manually optimized, the curves were iteratively computed with different values of the parameter and visually compared to the measured ones. When no further increase in matching could be obtained, the parameter was considered optimal. When using auto‐modeling, one parameter could be automatically optimized using profiles and/or PDDs of different field sizes and depths as input for the optimizer. Note that the input curves could come from only one collimator type even when the parameter influenced each model. In this case, a trade‐off had to be found. For fixed and Iris collimators, the modeling was performed independently for each collimator diameter.

**TABLE 1:** Parameters available for modeling in RayStation (RS)

Parameters	Collimator	Indicator	Manual/auto‐modeling
Energy spectrum	All	PDD	Auto‐modeling
Primary source	All	Steepness penumbra of small field sizes	Auto‐modeling
Flattening filter source	All	Out‐of‐field doses of large field sizes	Auto‐modeling
Energy correction factor	Fixed/Iris^a^	PDD	Manual
Diameter correction factor	Fixed/Iris	FWHM profile	Manual
Output correction factor	All	All	Auto‐modeling

Abbreviations: CC, collapsed cone; PDD, percentage depth doses.

^a^ The energy correction factor for the Iris collimator was not available for CC modeling.

The energy correction factors were not available for the Iris collimator for the CC algorithm (they were only available for MC). Therefore, only the output correction factors were used to optimize the PDDs. This was sufficient for diameters above 10 mm, but for smaller diameters (7.5 and 10 mm), no good correspondences could be obtained. Therefore, RS automatically disallowed these diameters for clinical use. In the event that these diameters have to be used clinically, the plan should be first optimized with CC and then recalculated with MC (because the MC optimization is not available).

The CC model was first created by optimizing the parameters mentioned in Table 1. After this, the MC model was calculated after duplication of the CC model and only a fine‐tuning of the parameters had to be performed.

The PDDs and profiles were optimized via a visual check of the superimposition of the measured and computed curves. When this was finished, the resulting models were quantitatively evaluated against the measurements with the following metrics:

- The mean dose difference (Δ*D*) at depths of 15, 50, 100, 150, and 200 mm on CAX (Δ*D* PDD).
- The FWHM difference (ΔFWHM) on LR‐profile measured at 100‐mm deep.

This analysis was performed for fixed and Iris collimators with beams of diameter of 7.5, 10, 15, 25, 35, and 50 mm (except the 7.5 and 10 mm for Iris in CC) and with beams of field size of 15.4 × 15.4, 30.8 × 30.8, 46.2 × 46.2, 53.8 × 53.9, and 84.6 × 84.7 mm^2^ for MLC.

### 2.2 Validation of collapsed cone and Monte Carlo models

For the model validation, we followed the recommendations made by the IAEA^7^ and AAPM^8^ that we adapted to CK and small fields dosimetry.

#### 2.2.1 Phantoms and dosimeters used for validation

**FIGURE 1:**
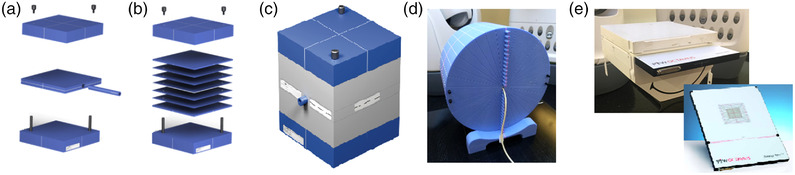
Phantoms used for dose measurement: (a) homogeneous phantom with A1SL insert (Phantom A), (b) homogeneous phantom with films insert (Phantom B), (c) heterogeneous phantom with a lung slab and A1SL insert (Phantom C), (d) homogeneous phantom with A1SL inserts in depth (Phantom D), and (e) Octavius phantom with PTW SRS 1000^12^ (Phantom E)

Validation measurements were performed using a set of homogeneous and heterogeneous phantoms presented in Figure 1. In the following, phantoms shown on Figure 1a–e are named Phantom A–E, respectively. Three different dosimeters metrologically traceable to the international standard of absorbed dose through the Swiss Federal Institute of Metrology (METAS) were used during the validation: the Exradin A1SL thimble ionization chamber (Standard Imaging, USA), EBT3 Gafchromic films (Ashland, USA), and a PTW 1000 SRS matrix of liquid filled ionization chambers (PTW, Germany).^9^ Because the 60018 diode was used for our model measurements (Section 2.1.1), we did not use it for the validation measurements to ensure an independent validation. These phantoms and detectors are described next.

Phantom A (Standard Imaging, USA) was used to measure doses for beams of diameter larger than 12.5 mm at 5‐cm deep in a homogenous medium, with the A1SL ionization chamber positioned on CAX. For diameters smaller than 12.5 mm, to avoid the volume‐averaging effect of the A1SL, EBT3 films were used in Phantom B (Standard Imaging, USA) in homogeneous condition. Indeed, EBT3 films are recommended for small fields’ dosimetry.^3^ In Phantom B, films were irradiated at 4.9‐cm deep. The films were previously calibrated with an Elekta Synergy linear accelerator (Elekta AB, Sweden) against a Farmer ionization chamber (Nuclear Enterprise, USA) traceable to international standard of absorbed dose through METAS with a 6‐MV beam energy. A ±4% (*k* = 2) uncertainty was considered for the dose estimation.^10^ To correct for background noise, nonirradiated films were also scanned, and the resulting background dose was subtracted from the irradiated films. The dose distributions calculated with RS were compared with the films using VeriSoft software (PTW, Germany) with a 3%/1‐mm global gamma index^11^ (GI). MEPHYSTO software (PTW, Germany) was used to extract LR‐profiles from the films and to compare their FWHM with calculated profiles.

The accuracy of the algorithms in heterogeneous conditions (low density of 0.26 g/cm^3^) was verified with Phantom C (Standard Imaging, USA). Measurements were performed with the A1SL ionization chamber at 10‐cm deep on CAX.

Phantom D (Accuray, USA) was employed to measure CAX dose at depths of 3, 6, 9, 15.5, and 19.5 cm with the A1SL ionization chamber.

Phantom E (PTW, Germany) was a half‐octavius phantom with an SRS 1000 array. This phantom was used for patient specific QAs as well as for off‐axis and irregular MLC beams verifications. It was also used to measure profiles for all collimators in order to compare the accuracy in the gradient regions between RS calculations and measurements.

The irradiation beams tested were designed either as a single‐beam (SB) with normal or oblique incidence on a phantom or with multiple‐beams (MB). For SB, a single‐aperture diameter was chosen and kept during the irradiation with the robot in a fixed position. During MB irradiation, either one or several aperture diameters were chosen with different angles of incidence for each beam. Before being compared to calculations, the measured doses were corrected for the daily output. The same procedure was followed to validate both the CC and the MC algorithms.

#### 2.2.2 Fixed collimator

The measurements performed to validate the fixed collimator are summarized in Table 2. We decided to focus on the smallest field sizes of 7.5 and 10 mm for the validation as we usually use those diameters when employing the fixed collimator. However, we also performed measurements with beams of 20‐ and 60‐mm diameter to validate larger field sizes.

**TABLE 2:** List of configurations tested for the validation of fixed, Iris, and multileaf collimators (MLC)

Configuration	Phantom	Irradiation type	Fixed diameter measured (mm)	Iris diameter measured (mm)	MLC field size measured (mm^2^)	Measured quantity
1	A	SB, normal incidence	20, 60	≥15	20 × 20, 60 × 60, 80 × 80	Δ*D*
2	B	SB, normal incidence	5, 7.5, 10, 12.5	For CC: 12.5 For MC: 7.5, 10, 12.5	–^a^	Δ*D* GI FWHM
3	A	SB, oblique incidence	20, 60	20, 40	–	Δ*D*
4	A/E	MB	20	Plan 1: 12.5, 15, 20 Plan 2: 20, 30, 40	20 × 20	Δ*D* Profiles
5	C	SB, normal incidence	20, 60	20, 40	40 × 45, 60 × 60	Δ*D*
6	C	SB, oblique incidence	60	20, 40	–	Δ*D*
7	C	MB	20	Plan 1: 20, 25, 30 Plan 2: 30, 40, 50	20 × 20	Δ*D*
8	D	Depth measurements	60	50, 60	38 × 60	Δ*D*
9	E	Patient QA	7.5	≥15	Irregular shapes	GI

Abbreviations: CC, collapsed cone; GI, gamma index; MB, multiple‐beam; MC, Monte Carlo; SB, single‐beam.

^a^ “–” Indicates that no measurements were performed for that configuration.

#### 2.2.3 Iris collimator

The configurations of measurements are presented in Table 2. Because we extensively use the Iris collimator with all diameters, all aperture sizes were tested for SB in a homogeneous phantom (configurations 1).

#### 2.2.4 MLC

**FIGURE 2:**
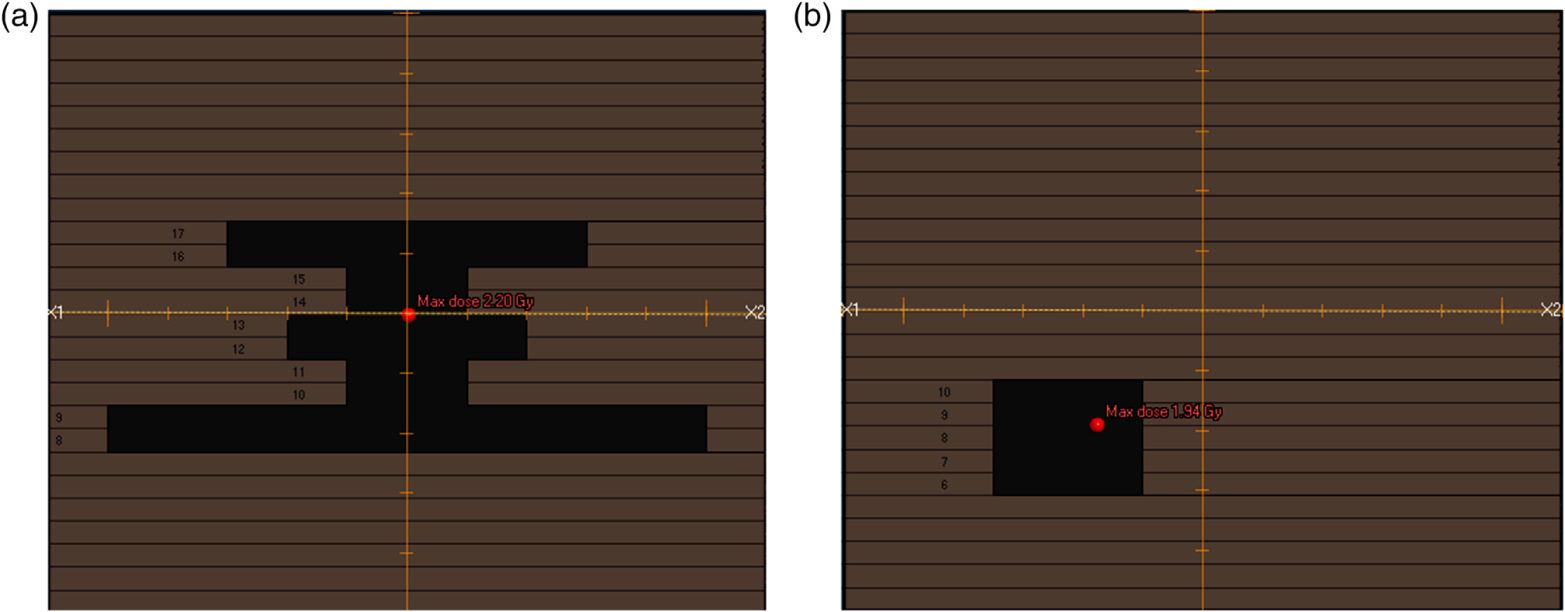
Beam eye's view of multileaf collimators (MLC) for (a) irregular field and (b) off‐axis field of beam *f*

The configurations used to validate the MLC are summarized in Table 2. In addition to these configurations, one irregular and several off‐axis beams were created in RS and measured with Phantom E (see Figure 2 for examples of beam's eye view). The comparison of measured and calculated dose was performed by a 3%/1‐mm GI. Off‐axis beams tested were shaped as

1. Symmetrical on *Y* (TG direction), open from *X* (LR direction) = 0 to *X* = 4 cm
2. Symmetrical on *Y*, open from *X* = 3 cm to *X* = 5 cm
3. Symmetrical on *Y*, open from *X* = −1 cm to *X* = −3 cm
4. Open from *Y* = 1 cm to *Y* = 3 cm, symmetrical on *X*
5. Open from *Y* = −1 cm to *Y* = −3 cm, symmetrical on *X*
6. Open from *Y* = −1 cm to *Y* = −3 cm, open from *X* = −1 cm to *X* = −3.5 cm

## 3 RESULTS

### 3.1 Collapsed cone and Monte Carlo modeling

The parameters described in Section 2.1.2 were straightforwardly optimized in RayPhysics. The PDDs and profiles computed with these parameters led to differences with the measurements within ±1%, as shown in Table 3.

**TABLE 3:** Differences obtained between computed and measured validation curves for all collimators and algorithms

	Fixed		Iris		MLC	
Algorithm	CC	MC	CC	MC	CC	MC
Δ*D* PDD (%)	0.0 ± 0.4	0.3 ± 0.5	0.1 ± 0.3	0.1 ± 0.5	0.1 ± 0.4	0.1 ± 0.3
ΔFWHM (%)	−0.3 ± 0.3	0.6 ± 0.6	0.0 ± 0.3	0.1 ± 0.3	−0.2 ± 0.2	0.1 ± 0.0

Abbreviations: CC, collapsed cone; MC, Monte Carlo; MLC, multileaf collimators; PDD, percentage depth doses.

### 3.2 Validation of the models

The Δ*D* obtained for configurations of Table 1 are summarized in Table 4.

**TABLE 4:** Dose differences obtained with measurements of the different configurations

Configuration		Fixed		Iris		MLC	
		CC	MC	CC	MC	CC	MC
1		−0.1 ± 0.0	0.2 ± 0.5	0.0 ± 0.5	0.4 ± 0.5	−0.1 ± 0.6	−0.2 ± 0.4
2		0.1 ± 1.4	−0.6 ± 1.9	−1.0^a^	0.9 ± 1.9	–^b^	–
3		−0.3 ± 0.9	−0.1 ± 0.5	−0.4 ± 0.1	0.7 ± 0.2	–	–
4		−0.1	−0.4	−0.8 ± 1.3	−0.4 ± 1.4	0.3	0.8
5		0.4 ± 0.3	−0.7 ± 0.7	0.4 ± 0.6	−0.5 ± 1.1	0.7 ± 0.2	0.4 ± 0.2
6		−0.6	0.1	−0.2 ± 0.1	0.0 ± 0.6	–	–
7		−0.7	−1.4	0.7 ± 0.1	−0.1 ± 0.4	0.6	−0.1
8	3 cm	0.3	−0.1	0.9	0.2	0.0	−0.5
	6 cm	0.5	0.5	−0.1	0.3	−0.2	−0.4
	9 cm	1.0	1.0	1.7	1.6	1.0	0.7
	15.5 cm	1.3	1.3	1.7	2.3	2.0	1.9
	19.5 cm	1.8	1.7	2.8	2.9	3.2	2.5
Average Δ*D* over configurations 1–8		0.3 ± 0.8	0.1 ± 0.9	0.5 ± 1.1	0.7 ± 1.1	0.8 ± 1.1	0.6 ± 1.0
9		100	100	98.2 ± 2.1	96.7 ± 2.1	99.5 ± 0.3	99.1 ± 0.5

Abbreviations: CC, collapsed cone; MC, Monte Carlo; MLC, multileaf collimators.

^a^ No standard deviation indicated when only one measurement was performed.

^b^ “–” Indicates that no measurements were performed for that configuration.

**FIGURE 3:**
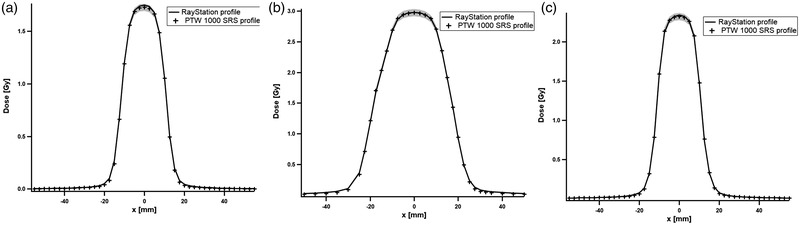
Comparison of profiles for multiple‐beam (MB) plan between the 1000 SRS detector (markers) and the RayStation (RS) treatment planning system (TPS) (filled line) calculated with collapsed cone (CC) algorithm with (a) fixed, (b) Iris, and (c) multileaf collimators (MLC). The gray area represents a dose difference of ±2%.

Figure 3 presents the comparison of MB plans irradiated on Phantom E and compared with the CC computed profile for each collimator. The comparison of profiles obtained with MC algorithm were similar (not shown).

Table 5 summarizes the results obtained from film dosimetry for fixed and Iris collimators and the GI obtained for irregular and off‐axis MLC beams.

**TABLE 5:** Results of film dosimetry (ΔFWHM and gamma index [GI]) for fixed and iris collimators and GI for irregular and off‐axis multileaf collimators (MLC) beams

	Collimator	Fixed		Iris		MLC	
	Algorithms	CC	MC	CC	MC	CC	MC
Film dosimetry (%)	ΔFWHM	−0.52 ± 1.08	−1.18 ± 0.73	−1.0	1.87 ± 0.51	–^a^	–
	GI	99.5 ± 0.6	99.4 ± 0.4	99.8	99.7 ± 0.2	–	–
GI Phantom E (%)	GI irregular field	–	–	–	–	100	98
	GI off‐axis beams	–	–	–	–	99.5 ± 0.8	98.8 ± 1.0

Abbreviations: CC, collapsed cone; MC, Monte Carlo.

^a^ Indicates that no measurements were performed for that configuration.

## 4 DISCUSSION

Both CC and MC models were created in RayPhysics in a straightforward manner. It was quite easy to optimize the energy spectrum using the auto‐modeling module. This optimization option also helped optimize the fluence parameters (primary and flattening filter sources), although they remained the hardest parameters to optimize because of their impact on all collimators. Indeed, the drawback of the auto‐modeling module for the source optimization was that it was impossible to choose profiles of different collimators to guide the optimization. Thus, only fixed, Iris, or MLC curves could be selected as the initial input of the optimization, which made it more difficult to find a good trade‐off. We used the MLC curves to perform the auto‐modeling of the fluence parameters and then manually adjusted these parameters to better match the fixed and Iris curves. The fixed and Iris collimators could be optimized without auto‐modeling. The absence of energy corrections factors in the CC algorithm for the Iris collimator had an impact on the 7.5‐ and 10‐mm diameters where no good correlation between the model and the measurements could be found. However, with the MC algorithm, the optimization of these field sizes worked well thanks to the energy correction factors and so has no direct influence in clinical use. That we were able to duplicate the CC model over to MC model was very useful because it offered a good starting point for the MC model. We found that for our purposes, the accuracy of the models was satisfactory.

The validation measurements confirmed the accuracy of the TPS calculations. We did not observe a better accuracy in one model compared to the other one, the averaged dose difference over all configurations being −0.06% ± 0.55% for both algorithms. The dose differences obtained in homogeneous and heterogeneous phantoms were comparable for both algorithms, demonstrating their accuracy in low‐density conditions. All configurations tested led to dose differences within ±1%, for all collimators and algorithms. There was one exception with configuration 8 (depth measurements) where dose differences increased up to 3.2% at 19.5‐cm deep. Moreover, we observed for all collimators and algorithms an increase of the dose difference with depth, which exceeded ±1% from 9‐cm deep and ±2% from 15.5‐cm deep. These results were nevertheless considered acceptable for clinical use.

To go a step further, we compared the dose differences obtained during MLC validation with the MC algorithm with a previous study performed on the MLC with the MC algorithm of Precision, where configurations 1, 4, 5, and 7 were tested with a similar method.^12^ The mean dose difference over these configurations was −1.3% ± 0.5% with the Precision TPS model, whereas 0.2% ± 0.5% with the RS MC model. Another study investigated the dose difference obtained with the previous Accuray TPS (MultiPlan) for fixed collimators and the MC algorithm, with configurations similar to configurations 1, 4, and 7.^13^ The mean dose difference obtained over all configurations was −0.1% ± 2.2% compared to −0.4% ± 0.6% for the RS. It is of note that in this study, dose differences with SB measurements on a homogeneous phantom were between −2.9% and 2.2%, whereas all dose differences were within ±1% with the RS.

The greatest challenge of the validation was the use of films for small field dosimetry because of the complexities involved in the accurate use of this dosimeter.^14^, ^15^, ^16^ Considering that the uncertainty involved in film dosimetry is higher than ionization chamber dosimetry, it was not possible to achieve an equivalent level of accuracy for beams with diameter under 12.5 mm compared to larger ones where an ionization chamber was used. Nevertheless, film measurements performed for the smallest field sizes of 7.5 and 10 mm made it possible for us to ensure that the absolute dose was within the uncertainty.

## 5 CONCLUSION

We performed straightforward modeling of CK collimators in the RS TPS for both CC and MC algorithms. The auto‐modeling module helped a lot during optimization of the models. The dose differences between measurements and the RS algorithms were within ±1% in most of the configurations and within ±3.5% for measurements as deep as 200 mm. In light of these results, we were able to validate both models for all collimators to be used clinically.

## AUTHOR CONTRIBUTION

All authors have made substantial contributions to conception and design, or acquisition of data, or analysis and interpretation of data, have been involved in drafting the manuscript or revising it critically for important intellectual content, have given final approval of the version to be published, have participated sufficiently in the work to take public responsibility for appropriate portions of the content, and have agreed to be accountable for all aspects of the work in ensuring that questions related to the accuracy or integrity of any part of the work are appropriately investigated and resolved.

## CONFLICT OF INTEREST

R. Moeckli is holding a grant from Accuray for a research in TomoTherapy. That grant is not related to the present study.

## Footnotes

1 RaySearch AB Laboratories, RayStation 11A Reference Manual, 2021.

2 RaySearch AB Laboratories, RayStation 11A Beam Commissioning Data Specification, 2021.
